# Discrete-Event Simulation Modeling in Healthcare: A Comprehensive Review

**DOI:** 10.3390/ijerph182212262

**Published:** 2021-11-22

**Authors:** Jesús Isaac Vázquez-Serrano, Rodrigo E. Peimbert-García, Leopoldo Eduardo Cárdenas-Barrón

**Affiliations:** 1 School of Engineering and Sciences, Tecnologico de Monterrey, Monterrey 64849, Northeast Nuevo Leon, Mexico; a01262327@itesm.mx (J.I.V.-S.); lecarden@tec.mx (L.E.C.-B.); 2 School of Engineering, Macquarie University, Sydney, NSW 2109, Australia

**Keywords:** discrete-event, simulation, modeling, healthcare, hospital, review, literature

## Abstract

Discrete-event simulation (DES) is a stochastic modeling approach widely used to address dynamic and complex systems, such as healthcare. In this review, academic databases were systematically searched to identify 231 papers focused on DES modeling in healthcare. These studies were sorted by year, approach, healthcare setting, outcome, provenance, and software use. Among the surveys, conceptual/theoretical studies, reviews, and case studies, it was found that almost two-thirds of the theoretical articles discuss models that include DES along with other analytical techniques, such as optimization and lean/six sigma, and one-third of the applications were carried out in more than one healthcare setting, with emergency departments being the most popular. Moreover, half of the applications seek to improve time- and efficiency-related metrics, and one-third of all papers use hybrid models. Finally, the most popular DES software is Arena and Simul8. Overall, there is an increasing trend towards using DES in healthcare to address issues at an operational level, yet less than 10% of DES applications present actual implementations following the modeling stage. Thus, future research should focus on the implementation of the models to assess their impact on healthcare processes, patients, and, possibly, their clinical value. Other areas are DES studies that emphasize their methodological formulation, as well as the development of frameworks for hybrid models.

## 1. Introduction

Healthcare systems are largely adaptive human-based systems that involve both the utilization of limited physical facilities and resources, and complex interactions among different healthcare groups [1,2,3]. Since these healthcare systems are characterized by a high level of variability and uncertainty, they are not naturally easy to understand, design, and predict [4,5,6,7].

As healthcare systems continually evolve, achieving better quality of care while reducing costs is a global concern [7,8]. Thus, strategic, tactical, and operational decisions are made daily to evaluate and improve the efficiency and effectiveness of different healthcare processes and services [3,7]. To foresee the impact of these decisions on the system performance, healthcare providers need proper tools, such as simulation, so they can effectively explore the alternative scenarios [1,9].

A simulation is an imitation of how the real-world system operates over time. This can be used to identify critical points and system bottlenecks, and to answer “what-if” questions about real-world scenarios without any practical and/or financial implications [10,11,12]. Simulations can estimate the consequences of different interventions in healthcare, allowing for the incorporation of behavioral aspects and personalized decisions [7], as well as for identifying the optimal scenario according to some output criteria [13].

A simulation study requires the definition of a conceptual model; a representation of a problem within a system that is derived from theory or observations [11,14,15]. This conceptual representation should integrate different components, such as objectives, inputs, outputs, content, boundaries, assumptions, and simplifications [16,17]. Later, the conceptual model is transferred into computer software that helps healthcare professionals to comprehend the relationship between the input and output variables of the real-world system [1,18].

Discrete-event simulation (DES), also referred to as a time-to-event model, is ideal for complex problems, such as healthcare ones [9,19]. DES is a computer-based operation research technique that models different systems as networks of queues and activities [18] in order to assess, predict, and optimize a proposed or existing system, where changes occur at discrete epochs over time [8,20,21,22]. DES emerged from the manufacturing world, wherein Tocher developed the first language in the late 1950s for constructing a model to simulate a steel plant in the UK [7,23]. DES is often used to represent systems at an operational level, where transactions, processes, and the flow of individual entities, as well as the variability, are important factors [4,24]. Hence, DES models use events and typical quantities to imitate the observed behavior of the system by generating deterministic quantities or stochastic distributions [3]. DES can capture a system´s behavior and interconnection effects, which result from the combinations of many random processes, coupled with the system structure [25]. Conversely, developing a DES model can be time consumingly (and costly), and it is heavily dependent on good quality data to inform the system behavior [24]. Users should, thus, balance the benefits and challenges of using the simulation approach.

The key concepts in DES are events, entities, attributes, and resources. An event is something that happens in the environment at a certain point in time. In the healthcare context, entities are self-contained objects that have attributes and consume resources while experiencing events, e.g., patients, organs for transplant, medical records, etc. [13,26]. Attributes are features or characteristics that are unique to an entity and can change over time, such as age and disease history, which influence their route through the simulation and the length of time between events [26]. Finally, resources are objects or facilities that provide a service to a dynamic entity, for example, doctors, nurses, hospital beds, operating rooms, physicians, etc. In addition, queues represent another important concept in DES, as they occur when several entities compete for a specific constrained resource, and they might have to wait until the resource is available. Each queue has its own logic and rules, commonly called a “queue discipline” [7,13,17].

Building a DES model requires large amounts of quantitative numerical data [18]. It also needs a set of logical statements that are expressed in a computable form to describe how the entities change their state [27]. DES has been used in healthcare as a preferable modeling technique, given its flexibility in responding to scale changes, the level of detail, individual patient focus, stochastic factors affecting the system, the ease in changing the model´s components, waiting for the time-related performance, the existence of queues, and the visual representation of patient flows [17]. Although big data analysis is emerging as a technique for data modeling and simulation, it presents more challenges in processes subject to changing conditions and unexpected events [28].

Table 1 summarizes the characteristics of discrete-event simulation. While DES outputs can be point estimates, as well as ranges of values, the experimental results can be measured in terms of performance metrics, such as resource utilization, waiting times, the number of entities in queues, and the throughput of services or products, among others [29].

As healthcare systems become more complex, in combination with stricter quality demands, there is also a growing interest in the use of DES modeling in these settings, exemplified by the increasing number of articles published in the literature every year (period 1994–2021). Since more than 200 research articles are found in the literature, this study conducts a comprehensive literature review to provide a wider perspective of the DES capabilities presented in healthcare until 2021. This paper provides a deep and detailed categorization of the DES articles in healthcare that will help researchers to identify the DES trends (areas of application, outcomes, software used, contribution of articles by country, and popular journals and publishers), and to identify opportunities for future research through four elements: the key elements to formulating models, frameworks for hybrid models, barriers for implementation, and measuring satisfaction and clinical value. The remainder of the article is presented as follows: Section 2 presents the methodology, including the search strategy, inclusion criteria, and review methodology. Section 3 presents the results and classification by year, approach, healthcare setting, outcome, provenance, and software use. Section 4 discusses these results, and Section 5 provides the conclusions.

## 2. Methods

### 2.1. Search Strategy

The databases Springer, BioMed Central, ScienceDirect, Web of Science, Research Gate, Wolters Kluwer, MDPI, Taylor & Francis, ProQuest, Wiley Online Library, Mary Ann Liebert, IEEE, Scopus, Emerald, Sage, BMJ, and PubMed Central were systematically searched to retrieve existing articles on DES applications in healthcare, until August 2021 when the last search was conducted. The key terms used to search included: “discrete event”, “DES”, “simulation”, “hospital”, and “healthcare”, in the title, abstract, and/or keywords. No restrictions related to year, approach, healthcare setting, outcome, country of provenance, or software use were considered.

### 2.2. Paper Inclusion Criteria

**Figure 1:**
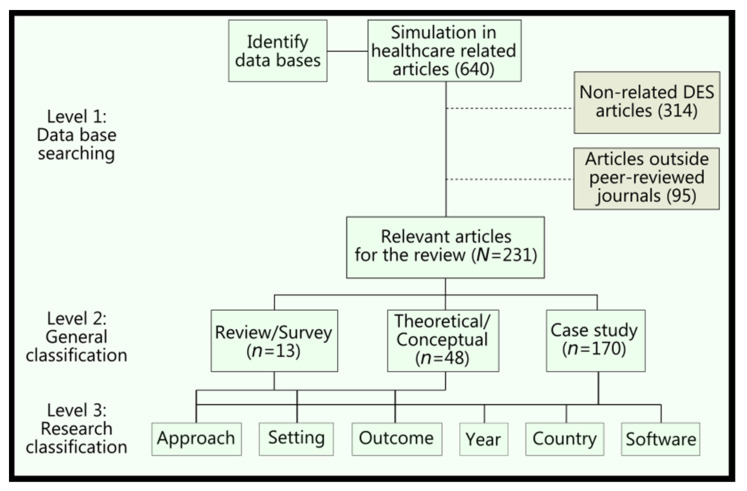
Inclusion and classification process of the review.

The inclusion criteria in this review were narrowed down to research articles that focus on DES in healthcare, including a range of studies from the exploration of theoretical aspects up to practical applications. Publications regarding other operational research techniques were excluded, but studies on hybrid DES models were included in this research. Non-English-language literature, and other English-language articles published outside peer-reviewed journals, such as conference papers, books, editor notes, etc., were discarded. Following the retrieval of publications, 231 papers were considered in this study. A total of 51.8% of the papers were retrieved from healthcare-related journals, while the rest were retrieved from industrial-engineering-related journals. Figure 1 shows the three-stage searching and sorting process that led to the research articles included in this study.

### 2.3. Review Methodology

The articles included in this review are divided into three taxonomy sections: (1) DES application articles that report original research; (2) Theoretical/conceptual articles that provide directions to explore problems or represent relations within DES models; and (3) Review articles that structure and classify the existing literature on the topic. Survey papers were analyzed alongside review papers as they were very few, and they focused on specific DES applications. Specific to the review papers included, only five studies focus entirely on DES as a unique review topic, and the rest aim to analyze healthcare improvements through diverse operations research techniques, DES being one of the approaches mentioned [21,30,31,32,33].

The search identified a total of 170 DES applications in healthcare, followed by 48 theoretical/conceptual articles, and 13 review/survey studies. Further classification within these main categories includes the approach, healthcare setting, outcome, country of provenance, and software use. While healthcare setting, country, and software use were directly extracted from the papers, the approach and outcome required deeper analysis. The review process also showed that approaches can vary, from unique DES applications up to models combining DES along with Markov models, Monte Carlo simulation (MCS), system dynamics (SD), agent-based simulation (ABS), optimization (Opt), mathematical models (Math models), and lean/six sigma. Specific to the review and theoretical/conceptual papers, the empirical outcomes, defined after the analysis of the papers, are the descriptions of the operational research techniques, the descriptions of the healthcare backgrounds, and the frameworks. Likewise, possible outcomes for DES application papers are:

- Time and efficiency;
- Financial and cost savings;
- Allocation of resources/schedule;
- Quality and defects;
- Patient health/safety.

## 3. Results

**Figure 2:**
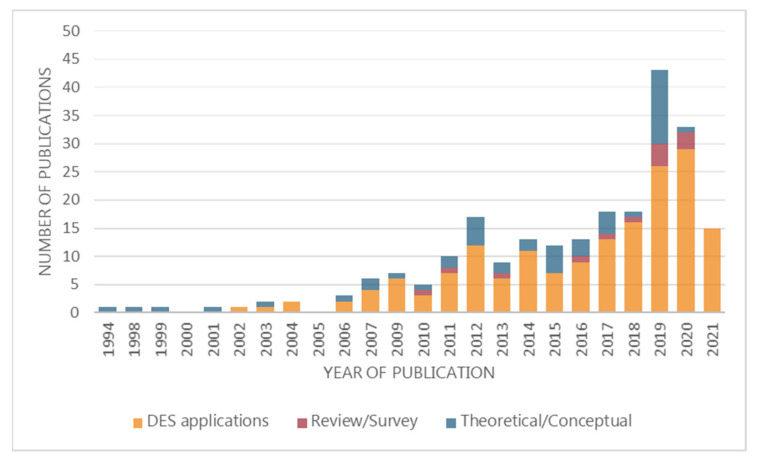
Number of publications per year.

This section is presented through the three taxonomies mentioned before: (1) Review papers; (2) Theoretical/conceptual papers; and (3) DES application papers. Figure 2 presents the distribution of publications over the years.

### 3.1. Review and Survey Papers

Review papers are characterized by the exploration and classification of DES developments in healthcare, and commonly utilize descriptive statistics and frequency counting. DES-related reviews generally analyzed the general healthcare domain (80%), while the rest analyzed applications on a specific area or application. Moreover, half of the paper-reviewed studies consider DES in combination with several other techniques. Table 2 presents the complete set/approach classification and percentages of the review papers considered.

The main limitation of previous DES-related reviews in healthcare is the narrow scope and contribution; some of them focus only on a specific taxonomy or study type, while others do not consider hybrid models, or they divide a shallow classification into fewer categories. Finally, the current directions for future research are very limited since the research was conducted some time ago.

### 3.2. Theoretical/Conceptual Papers

The aim of the theoretical/conceptual papers is mainly to provide support for performing practical DES applications in healthcare. Developing DES theory and the concepts within healthcare are focused on emergency departments in 13% of the cases, and on the general healthcare domain in 36% of the cases, as per Table 3. Frameworks for the DES applications are provided in 44% of the studies, and the use of DES hybrid models is discussed in 63% of these.

### 3.3. DES Applications Papers

#### 3.3.1. Approach

**Figure 3:**
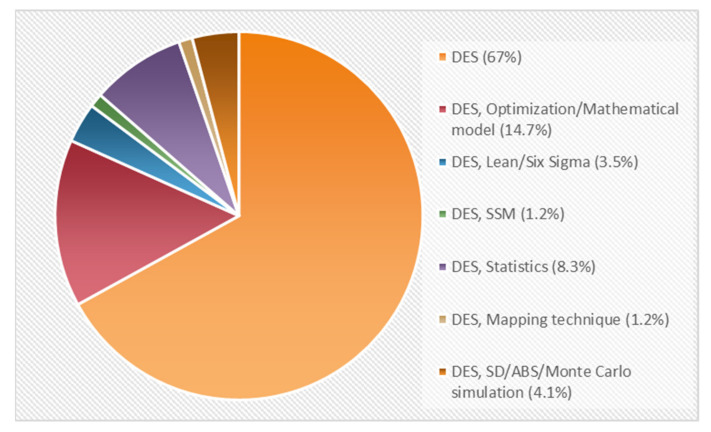
Approaches of applied research papers.

All 170 DES models were validated, and different “what-if” scenarios have been tested with each model. However, less than 10% have carried out implementation to improve the system’s performance (it was considered that a study had an actual implementation if that is stated in the paper, or if evidence of implementation is shown). On the other hand, one-third of the DES applications are complemented with another technique, such as operations research, so they can provide a wider range of characteristics to solve operational healthcare problems. Other hybrid models combine DES with different simulation approaches, such as system dynamics (SD), agent-based simulation (ABS), and Monte Carlo simulation (MCS), in order to complement and enlarge the scope, purpose, and perspective of the simulation. Inferential statistics are also considered and used to infer and make concise predictions about the indicators used in the simulation. Moreover, the soft systems methodology (SSM) is also incorporated to justify changes and/or improvements in organizational systems. Other approaches include optimization models to mathematically describe those factors that are not explained only with probability distributions, and lean/six sigma, and/or mapping techniques to improve the system under study. Figure 3 shows the percentages of the approaches used in the studies.

#### 3.3.2. Setting and Outcomes

A total of 38.9% of the DES application studies were conducted in hospital and medical centers, while 21.8% specifically focused on emergency departments, and 13% on the patient clinical conditions. Moreover, half of the outcomes reported in these studies are related to time and efficiency, 21.2% to the allocation of resources/schedules, and 12.3% on financial and cost savings. Table 4 presents the classification of papers based on the healthcare setting under study and the corresponding outcomes.

#### 3.3.3. Journals, Publishers, and Countries

The journals with the most DES publications in healthcare are *Health Care Management Science* (6% of papers. Rank 2020: SJR 0.9, Q1; CiteScore Scopus 4.6), the *Journal of the Operational Research Society* (5% of papers. Rank 2020: SJR 0.753, Q2; CiteScore Scopus 4.1), and the *Journal of Simulation* (4%. Rank 2020: SJR 0.294, Q3; CiteScore Scopus 3.5). Meanwhile, the top publishers are Elsevier (20%), Springer (20%), and Taylor & Francis (10%). Table 5 presents the top ten publications by the number of citations, as retrieved from Scopus in October 2021.

Concerning countries where these DES-related studies were carried out, 26% of the publications proceeded from authors with affiliations in the US, 19% from the UK, and 12% from Canada. In contrast, the top developing countries, such as Brazil, Egypt, and Malaysia, have each contributed to 4% of the literature. Table 6 shows the main publishers and countries in the literature. Before 2012, almost 50% of the studies were published by institutions affiliated with the U.S. and the U.K. As of 2012, the application of DES in the health sector has become widespread throughout the world.

#### 3.3.4. Software Use

Specialized DES software is used in 88% of the articles, whereas the complementary 12% utilized low-level simulation scripting languages, such as Python, or intermediate-level simulation tools that incorporated low-level scripting with enhanced graphic interfaces, such as MATLAB (MathWorks, Natick, MA, USA) and Visual Object Net++ (Dr. Reiner Drath, Illemnau, Germany) [235]. The reason why specialized DES software is used the most is that it provides the modeler with an environment that, in comparison to scripting languages, allows for the creation of models in less time and with less complexity. The most common software is Arena (Rockwell Automation, Milwaukee, WI, USA) (35%) and Simul8 (Simul8 Corporation, Boston, MA, USA) (21%). Within articles presenting hybrid models, around half use a specialized DES software, while Arena remains the most used software (22%). However, 32% of the publications do not mention the software utilized.

## 4. Discussion

The popularity of DES in healthcare is notably increasing, as almost 40% of the papers were published in the last three years. This is due to its ability to include high levels of detail and the ease-of-modeling medical processes using stochastic factors. Lately, DES is being applied in emergency departments, where short lead times and the efficient use of resources are key to operating. Similarly, the clinical analysis of entities (patient clinical condition) is emerging as a broader perspective from which to apply DES from a strictly medical perspective (13% of the application papers). The simulation of the clinical condition of patients plays a critical role in reducing treatment costs, improving the efficiency in the use of medicines, and analyzing the medical evolution of patients out of acute care.

Even though three countries concentrate 57.7% of the publications addressing DES in healthcare (the US, the UK, and Canada), the fundamental tools for engaging the stakeholders in healthcare systems worldwide in the development and application of DES are the virtual interaction elements, such as user interfaces. In addition, the software used to carry out simulations plays an essential role in the DES involvement in healthcare. A specific and flexible DES software has a higher probability of adapting to the healthcare stakeholders’ needs. This is the reason why only 12% of the papers utilize low-level simulation scripting languages.

**Figure 4:**
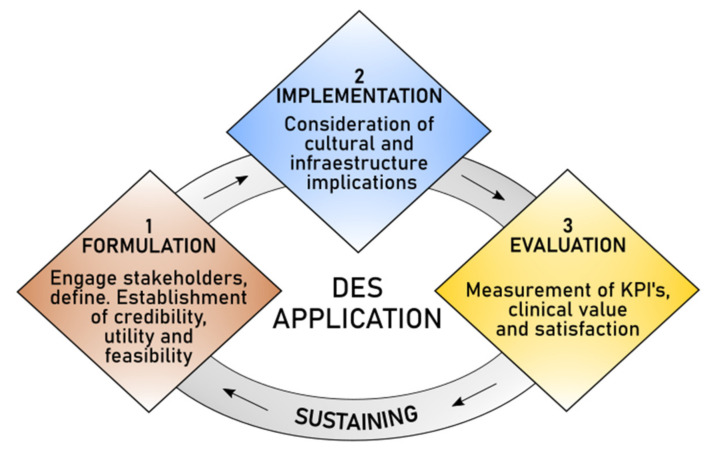
Holistic perspective for DES applications in healthcare.

Several elements have caused the impact of DES on healthcare improvement to be questioned, such as the limited scope of the studies found in the literature, and the contextual factors that make healthcare improvement complex. Thus, this discussion is presented through four main areas that present opportunities for further research: key elements to formulate models, frameworks for hybrid models, barriers for implementation, and measuring satisfaction and clinical value. Figure 4 presents the perspective of DES in healthcare considering these elements.

### 4.1. Key Elements to Formulate Models

The formulation of a model plays a critical role in simulation research, as it ensures that the modeler depicts the right theoretical state and focuses the impact on the root causes of the problem. A good formulation should consider five key elements: stakeholder engagement, definition, credibility, utility, and feasibility [8,53,65,114]. When theory and applications are supported by a proper formulation, publications tend to be beneficial for both researchers and system stakeholders.

Despite some theoretical publications addressing stakeholder engagement, this is not usually considered in DES applications. Engaging all stakeholders is key to formulating a simulation project [53], particularly in the healthcare context, where there is a plurality of stakeholder opinions, objectives, and power distributions [114]. In conjunction with stakeholders, modelers should define the causes of the problem, the main goal sought, and the internal and external influences that intervene in defining that goal [8]. In alliance with the system’s stakeholders, it should be defined whether the conceptual model is sufficiently accurate for the purpose at hand (credibility), if it assists decisionmakers in the problem situation (utility), and if any project limitations, such as time, resources, and/or data availability, are considered (feasibility) [65]. Then, conducting more studies built over these formulation elements are required.

### 4.2. Frameworks for Hybrid Models

Given that healthcare systems are complex, there are a plethora of problems that cannot be analyzed using a single method. Hybrid approaches provide a more realistic picture of complex systems with fewer assumptions and less complexity [9], which, in turn, allows for addressing a larger range of modeling questions [74]. There is specialized software, such as Simul8, AnyLogic, and Arena that allow the modeler to develop hybrid simulation models in the same interface/environment, as developed in the research presented in [236,237]. However, in the healthcare context, combining simulation techniques is not enough; even when this review has shown that hybrid DES models (mathematical models, statistics, improvement methodologies, or mapping techniques) have been broadly applied over the last years, there are no frameworks available that can serve as the foundation for successful modeling and implementation. It is important to have this kind of structure that can guide the modeler in developing more robust hybrid models. Additionally, a framework should provide support for identifying the object or system (What), the purpose (Why), and the methodology (How) [74]. Moreover, it should allow for the recognition of the correct approach/technique for collecting data and evaluating the long-term effects and outcomes [9].

### 4.3. Barriers for Implementation

A proper formulation does not guarantee that DES models will be implemented. Furthermore, transformation efforts never come without challenges [197]. Although simulation is widely reported upon in healthcare, it is not clear whether there is an actual implementation and impact in the real health system [23,238]. It was found that less than 10% of studies showed evidence of implementation. Most of the DES models applied to healthcare settings are led by academics, mainly for research purposes, and they have a limited impact on the potential performance of the systems [23]. Two major barriers to implementation have been identified in this study. First, there is the cultural side, as healthcare professionals (e.g., doctors and nurses) respond to pressure and system modifications by changing their performance and behavior [9]. In conjunction with changes, diversity across entities causes a lack of acceptance and fear regarding information and confidentiality [229]. Second, infrastructure plays a critical role. Difficulty in accessing enough quality data, system failures, and changes in work processes, security, and privacy, all are critical barriers to implementing models [229]. In addition, other financial constraints could undermine research.

Because of the diversity of health systems, no panacea for implementation exists. However, future research should reach to the models’ implementations and follow through after the intervention [29] in order to evaluate the long-term effects. This would convince service providers and clinicians that simulation can make a critical contribution [46].

### 4.4. Measuring Satisfaction and Clinical Value

Most DES applications in healthcare focus on improving direct metrics, such as volume, efficiency, and occupancy rates, whereas after-implementation metrics related to patient satisfaction and value are more difficult and less common. High levels of value and patient satisfaction are associated with better outcomes, given that satisfied patients are more likely to adhere to treatment. Conversely, low patient satisfaction affects treatment compliance, including return visit rates [22]. Thus, measuring these levels is a challenging task [132], and developing methods/techniques alongside surveys and questionaries to measure them represents a gap in the advances of DES applications in healthcare [230].

## 5. Conclusions

DES is a stochastic approach that is becoming more popular. This is reflected in the growing number of research articles that are focused on DES in healthcare. A descriptive analysis of DES publications in healthcare was conducted in this study to identify both current trends in research and directions for future research. The findings show a tendency to use this approach within emergency departments, patient clinical conditions, and medical centers seeking to allocate resources and improve times and efficiency. The results also indicate that the main issues addressed through DES are related to operations, where there is a need for high levels of efficiency and financial savings. The US, the UK, and Canada are the top countries that continually look towards improving their healthcare systems, as per Table 5. It was also found that the most popular DES software for the studies is Arena.

The large number of papers considered for this review (231) have shown the versatility of the DES approach, as well as the broad adoption of operational research techniques within some healthcare systems. Even though 231 papers is a large number, it represents a small proportion of the papers presenting analytical studies in healthcare. Healthcare is an area where researchers focus on the application of operations research techniques; however, DES is not being applied as much as lean/six sigma and other optimization techniques. Specific to hybrid approaches, the combination of several techniques can create a solid analytical approach that addresses the weaknesses of DES, such as strategic alignment and stakeholder behavior, as well as integrated levels.

DES models formulated in future research need to tackle two elements: proper and correct formulation, and the incorporation of the behavior of healthcare staff, in order to defeat cultural obstacles. Furthermore, researchers and professionals should define key infrastructural and financial capacities. Finally, the evaluation of the long-term effects, along with the publication of successful implementations following DES modeling, are key opportunities that need to be addressed in future DES-related research in healthcare.

## Figures and Tables

**Table 1:** Characteristics of discrete event simulation.

Scope:	Operational, tactical
Purpose:	Decisions: Optimizations, predictions, and comparisons
Perspective:	Analytic, emphasis on detail complexity
Importance of variability:	High
Importance of tracking individuals:	High
Number of entities:	Large
Control:	Waiting (queues)
Relative timescale:	Short
Resolution of models:	Individual entities, attributes, decisions, and events
Data sources:	Numeric with some critical elements
Lower boundary of technical preparation:	Qualitative workflow
Model elements:	Physical, tangible, information
Model outputs:	Prediction points, performance measurements
Tools:	Arena, Simul8, FlexSim/FlexSim Healthcare, ProModel/MedModel, Simio, AnyLogic, TreeAge, ExtendSim

**Table 2:** Discrete-event simulation review papers in the literature.

Setting/Approach	DES	DES + Markov	DES + SD or ABS	DES + Others	Total
General Healthcare	[30,31,32,33,34,35]	[36]	[37]	[38,39]	10
Emergency Unit				[40]	1
Medical Center	[21]		[41]		2
Total	7	1	2	3	13

Survey papers: [34,35,36,37,38,39,40,41]

**Table 3:** Discrete-event simulation theoretical and conceptual papers in the literature.

Setting/Approach	DES	DES + Optimization or Math Model	DES + Lean or Six Sigma	DES + SD or ABS or Monte Carlo	DES + Others	Total	%
General Healthcare	[42,43,44,45]	[20]		[3,15,18,43,46,47,48,49]	[26,50,51,52]	17	36
Emergency Unit	[53,54,55,56]	[57]		[9]		6	13
Intensive Unit					[58]	1	2
Operating Room			[22]			1	2
Pediatric	[13]					1	2
Therapy				[24,59]		2	4
Psychiatry				[60]		1	2
Patient State	[61,62,63,64]			[65]		5	10
Medical Center	[66,67,68,69,70]	[2,71,72]	[73]	[7,17,74,75,76]		14	29
Total	18	5	2	18	5	48	
%	38	10	4	38	10		100

**Table 4:** Discrete-event simulation publications classified by setting and outcome.

Setting/Outcome	Time and Efficiency	Financial and Cost Savings	Allocation of Resources/Schedule	Public Health	Others	Total	%
Clinic		[77,78]		[79,80]		4	2.3
Emergency Unit	[10,11,81,82,83,84,85,86,87,88,89,90,91,92,93,94,95,96,97,98,99,100,101,102,103,104]		[105,106,107,108,109,110,111,112,113]		[1,114]	37	21.8
Intensive Unit	[115,116,117]		[118]			4	2.4
Laboratory	[16]					1	0.5
Nursing				[119]	[8]	2	1.2
Oncology	[120,121]	[122,123]				4	2.4
Operating Room	[124,125,126]		[127,128]			5	3
Orthopedic	[129,130,131]	[132]	[6,133]			6	3.6
Pathology			[134]			1	0.5
Pediatric			[135]			1	0.5
Therapy	[136,137]					2	1.2
Pharmacy	[138,139]					2	1.2
Radiology	[140,141,142,143,144]					5	3
Support Areas	[145]		[146,147,148]		[149]	5	3
Dental Area	[150]					1	0.5
Mammography	[151]					1	0.5
Patient State	[152]	[19,153,154,155,156,157,158,159,160,161,162,163]		[164,165,166,167,168,169,170,171,172]		22	13
Medical Device				[173]		1	0.5
Medical Center	[12,14,22,27,174,175,176,177,178,179,180,181,182,183,184,185,186,187,188,189,190,191,192,193,194,195,196,197,198,199,200,201,202]	[203,204,205,206]	[207,208,209,210,211,212,213,214,215,216,217,218,219,220,221,222,223]	[25,224,225,226,227,228]	[229,230,231,232,233,234]	66	38.9
Total	84	21	36	19	10	170	
%	49.4	12.3	21.2	11.2	5.9		100

**Table 5:** Top DES publications by number of citations.

Article	Journal	Publisher	Number of Citations	Publication Year	Average Citations per Year (until 2021)
[182]	*Health Care Management Science*	Springer	117	2006	7.8
[131]	*Health Care Management Science*	Springer	113	2011	11.3
[204]	*Health Economics*	Wiley	100	2003	5.6
[233]	*Health Care Management Science*	Springer	69	2002	3.6
[25]	*European Journal of Operational Research*	Elsevier	67	2014	9.6
[149]	*Health Care Management Science*	Springer	66	2007	4.7
[184]	*Production and Operations Management*	Wiley	57	2011	5.7
[125]	*Health Care Management Science*	Springer	55	2010	5.0
[87]	*Simulation Modelling Practice and Theory*	Elsevier	50	2015	8.3
[121]	*European Journal of Operational Research*	Elsevier	49	2016	9.8

**Table 6:** DES publications classified by main publishers and countries.

Country/Publisher	Springer	Elsevier	Taylor & Francis	Palgrave	Others	Total	%
US	11	8	5	0	20	44	25.9
UK	5	4	4	9	11	33	19.4
Canada	4	4	3	1	9	21	12.4
Others	14	18	6	3	31	72	42.3
Total	34	34	18	13	71	170	
%	20	20	10.5	7.7	41.8		100

## Data Availability

Not applicable.

## References

[1] Thorwarth M., Rashwan W., Arisha A. (2016). An analytical representation of flexible resource allocation in hospitals. Flex. Serv. Manuf. J..

[2] Ben-Tovim D., Filar J., Hakendorf P., Qin S., Thompson C., Ward D. (2016). Hospital Event Simulation Model: Arrivals to Discharge–Design, development and application. Simul. Model. Pract. Theory.

[3] Chahal K., Eldabi T. (2011). Hybrid simulation and modes of governance in UK healthcare. Transform. Gov. People Process Policy.

[4] Koelling P., Schwandt M.J. Health systems: A dynamic system—Benefits from system dynamics. Proceedings of the Winter Simulation Conference.

[5] Arisha A., Rashwan W. (2016). Modeling of healthcare systems: Past, current and future trends. Proc. Winter Simul. Conf..

[6] Gillespie J., McClean S., FitzGibbons F., Scotney B., Dobbs F., Meenan B.J. (2014). Do we need stochastic models for healthcare? The case of ICATS?. J. Simul..

[7] Marshall D.A., Burgos-Liz L., Ijzerman M.J., Osgood N.D., Padula W.V., Higashi M.K., Wong P.K., Pasupathy K.S., Crown W. (2015). Applying dynamic simulation modeling methods in health care delivery research—The SIMULATE checklist: Report of the ISPOR simulation modeling emerging good practices task force. Value Health.

[8] Qureshi S.M., Purdy N., Mohani A., Neumann W.P. (2019). Predicting the effect of nurse–patient ratio on nurse workload and care quality using discrete event simulation. J. Nurs. Manag..

[9] Chahal K., Eldabi T., Young T. (2013). A conceptual framework for hybrid system dynamics and discrete event simulation for healthcare. J. Enterp. Inf. Manag..

[10] Landa P., Sonnessa M., Tànfani E., Testi A. (2018). Multiobjective bed management considering emergency and elective patient flows. Int. Trans. Oper. Res..

[11] Hajjarsaraei H., Shirazi B., Rezaeian J. (2018). Scenario-based analysis of fast track strategy optimization on emergency department using integrated safety simulation. Saf. Sci..

[12] Stahl J.E., Roberts M.S., Gazelle S. (2003). Optimizing management and financial performance of the teaching ambulatory care clinic. J. Gen. Intern. Med..

[13] Ramwadhdoebe S., Buskens E., Sakkers R.J.B., Stahl J.E. (2009). A tutorial on discrete-event simulation for health policy design and decision making: Optimizing pediatric ultrasound screening for hip dysplasia as an illustration. Health Policy.

[14] Abubakar A.M., Adamu A., Abdulkadir A., Abdulkadir H.S. (2020). Discrete Event Simulation of Clients Flow in Ante-natal Clinic. Asian J. Probab. Stat..

[15] Weinstein M.C. (2006). Recent developments in decision-analytic modelling for economic evaluation. Pharmacoeconomics.

[16] Pongjetanapong K., O’Sullivan M., Walker C., Furian N. (2018). Implementing complex task allocation in a cytology lab via HCCM using Flexsim HC. Simul. Model. Pract. Theory.

[17] Gunal M.M. (2012). A guide for building hospital simulation models. Health Syst..

[18] Brailsford S., Hilton N. (2001). A comparison of discrete event simulation and system dynamics for modelling health care systems. Proc. ORAHS 2000.

[19] Raphael J., Helou J., Pritchard K.I., Naimark D.M. (2017). Palbociclib in hormone receptor positive advanced breast cancer: A cost-utility analysis. Eur. J. Cancer.

[20] Dehghanimohammadabadi M., Keyser T.K. (2017). Intelligent simulation: Integration of SIMIO and MATLAB to deploy decision support systems to simulation environment. Simul. Model. Pract. Theory.

[21] Zhang X. (2018). Application of discrete event simulation in health care: A systematic review. BMC Health Serv. Res..

[22] Lenin R.B., Lowery C.L., Hitt W.C., Manning N.A., Lowery P., Eswaran H. (2015). Optimizing appointment template and number of staff of an OB/GYN clinic—Micro and macro simulation analyses. BMC Health Serv. Res..

[23] Robinson S., Radnor Z.J., Burgess N., Worthington C. (2012). SimLean: Utilising simulation in the implementation of lean in healthcare. Eur. J. Oper. Res..

[24] Yip K.C.M., Huang K.W.H., Ho E.W.Y., Chan W.K., Lee I.L.Y. (2017). Lessons from mixing OR methods in practice: Using DES and SD to explore a radiotherapy treatment planning process. Health Syst..

[25] Viana J., Brailsford S.C., Harindra V., Harper P.R. (2014). Combining discrete-event simulation and system dynamics in a healthcare setting: A composite model for Chlamydia infection. Eur. J. Oper. Res..

[26] Cooper K., Brailsford S.C., Davies R. (2007). Choice of modelling technique for evaluating health care interventions. J. Oper. Res. Soc..

[27] Fialho A.S., Oliveira M.D., Sá A.B. (2011). Using discrete event simulation to compare the performance of family health unit and primary health care centre organizational models in Portugal. BMC Health Serv. Res..

[28] Kim B.S., Kang B.G., Choi S.H., Kim T.G. (2017). Data modeling versus simulation modeling in the big data era: Case study of a greenhouse control system. Simulation.

[29] Marshall D.A., Burgos-Liz L., Ijzerman M.J., Crown W., Padula W.V., Wong P.K., Pasupathy K.S., Higashi M.K., Osgood N.D. (2015). Selecting a dynamic simulation modeling method for health care delivery research—Part 2: Report of the ISPOR dynamic simulation modeling emerging good practices task force. Value Health.

[30] Günal M.M., Pidd M. (2010). Discrete event simulation for performance modelling in health care: A review of the literature. J. Simul..

[31] Karnon J., Haji Ali Afzali H. (2014). When to use Discrete Event Simulation (DES) for the economic evaluation of health technologies? A review and critique of the costs and benefits of DES. Pharmacoeconomics.

[32] Zhang X., Lhachimi S.K., Rogowski W.H. (2020). Reporting Quality of Discrete Event Simulations in Healthcare—Results from a Generic Reporting Checklist. Value Health.

[33] Liu S., Li Y., Triantis K.P., Xue H., Wang Y. (2020). The Diffusion of Discrete Event Simulation Approaches in Health Care Management in the Past Four Decades: A Comprehensive Review. MDM Policy Pract..

[34] Hvitfeldt-Forsberg H., Mazzocato P., Glaser D., Keller C., Unbeck M. (2017). Staffs’ and managers’ perceptions of how and when discrete event simulation modelling can be used as a decision support in quality improvement: A focus group discussion study at two hospital settings in Sweden. BMJ Open.

[35] Alrabghi A. (2020). Simulation in saudi healthcare: An empirical study revealing the current status and future prospects. South Afr. J. Ind. Eng..

[36] Standfield L., Comans T., Scuffham P. (2014). Markov modeling and discrete event simulation in health care: A systematic comparison. Int. J. Technol. Assess. Health Care.

[37] Zhang C., Grandits T., Härenstam K.P., Hauge J.B., Meijer S. (2019). A systematic literature review of simulation models for non-technical skill training in healthcare logistics. Adv. Simul..

[38] Salleh S., Thokala P., Brennan A., Hughes R., Booth A. (2017). Simulation Modelling in Healthcare: An Umbrella Review of Systematic Literature Reviews. Pharmacoeconomics.

[39] Hughes V.S., Ferreira De Azeredo-Da-Silva A.L., Hincapie A.L. (2019). Health Economics and Outcomes Research (HEOR) Knowledge Needs Assessment for Latin America. Value Health Reg. Issues.

[40] Paul S.A., Reddy M.C., Deflitch C.J. (2010). A systematic review of simulation studies investigating emergency department overcrowding. Simulation.

[41] Katsaliaki K., Mustafee N. (2011). Applications of simulation within the healthcare context. J. Oper. Res. Soc..

[42] Mustafee N., Taylor S., Katsaliaki K., Dwivedi Y., Williams M. (2012). Motivations and barriers in using distributed supply chain simulation. Int. Trans. Oper. Res..

[43] Jun J.B., Jacobson S.H., Swisher J.R. (1999). Application of discrete-event simulation in health care clinics: A survey. J. Oper. Res. Soc..

[44] Komashie A., Mousavi A., Gore J. (2007). Quality management in healthcare and industry: A comparative review and emerging themes. J. Manag. Hist..

[45] Fletcher A., Worthington D. (2009). What is a “generic” hospital model?—A comparison of “generic” and “specific” hospital models of emergency patient flows. Health Care Manag. Sci..

[46] Eldabi T., Paul R.J., Young T. (2007). Simulation modelling in healthcare: Reviewing legacies and investigating futures. J. Oper. Res. Soc..

[47] Tako A.A., Kotiadis K. (2015). PartiSim: A multi-methodology framework to support facilitated simulation modelling in healthcare. Eur. J. Oper. Res..

[48] Maidstone R. (2012). Discrete Event Simulation, System Dynamics and Agent Based Simulation: Discussion and Comparison. System.

[49] Bayer S. (2014). Simulation modelling and resource allocation in complex services. BMJ Qual. Saf..

[50] Caro J.J., Möller J., Getsios D. (2010). Discrete event simulation: The preferred technique for health economic evaluations?. Value Health.

[51] Jun G.T., Morris Z., Eldabi T., Harper P., Naseer A., Patel B., Clarkson J.P. (2011). Development of modelling method selection tool for health services management: From problem structuring methods to modelling and simulation methods. BMC Health Serv. Res..

[52] Abe T.K., Beamon B.M., Storch R.L., Agus J. (2016). Operations research applications in hospital operations: Part III. IIE Trans. Healthc. Syst. Eng..

[53] Bean D.M., Taylor P., Dobson R.J.B. (2019). A patient flow simulator for healthcare management education. BMJ Simul. Technol. Enhanc. Learn..

[54] Ahalt V., Argon N.T., Ziya S., Strickler J., Mehrotra A. (2018). Comparison of emergency department crowding scores: A discrete-event simulation approach. Health Care Manag. Sci..

[55] Hamrock E., Paige K., Parks J., Scheulen J., Levin S. (2013). Discrete event simulation for healthcare organizations: A tool for decision making. J. Healthc. Manag..

[56] Bowers J., Ghattas M., Mould G. (2012). Exploring alternative routes to realising the benefits of simulation in healthcare. J. Oper. Res. Soc..

[57] Enayati S., Mayorga M.E., Rajagopalan H.K., Saydam C. (2018). Real-time ambulance redeployment approach to improve service coverage with fair and restricted workload for EMS providers. Omega.

[58] Dong Y., Chbat N.W., Gupta A., Hadzikadic M., Gajic O. (2012). Systems modeling and simulation applications for critical care medicine. Ann. Intensive Care.

[59] Brown J., Karnon J. (1998). Selecting a decision model for economic evaluation: A case study and review. Health Care Manag. Sci..

[60] Langellier B.A., Yang Y., Purtle J., Nelson K.L., Stankov I., Diez Roux A.V. (2019). Complex Systems Approaches to Understand Drivers of Mental Health and Inform Mental Health Policy: A Systematic Review. Adm. Policy Ment. Health Ment. Health Serv. Res..

[61] Caro J.J., Huybrechts K.F., Xenakis J.G., O’Brien J.A., Rajagopalan K., Lee K. (2006). Budgetary impact of treating acute bipolar mania in hospitalized patients with quetiapine: An economic analysis of clinical trials. Curr. Med. Res. Opin..

[62] Degeling K., Koffijberg H., Franken M.D., Koopman M., IJzerman M.J. (2019). Comparing Strategies for Modeling Competing Risks in Discrete-Event Simulations: A Simulation Study and Illustration in Colorectal Cancer. Med. Decis. Mak..

[63] Kotiadis K., Tako A.A. (2018). Facilitated post-model coding in discrete event simulation (DES): A case study in healthcare. Eur. J. Oper. Res..

[64] Jahn B., Theurl E., Siebert U., Pfeiffer K.P. (2010). Tutorial in medical decision modeling incorporating waiting lines and queues using discrete event simulation. Value Health.

[65] Kotiadis K., Tako A.A., Vasilakis C. (2014). A participative and facilitative conceptual modelling framework for discrete event simulation studies in healthcare. J. Oper. Res. Soc..

[66] Pinha D.C., Ahluwalia R.S. (2019). Flexible resource management and its effect on project cost and duration. J. Ind. Eng. Int..

[67] Brazier J., Ara R., Azzabi I., Busschbach J., Chevrou-Séverac H., Crawford B., Cruz L., Karnon J., Lloyd A., Paisley S. (2019). Identification, Review, and Use of Health State Utilities in Cost-Effectiveness Models: An ISPOR Good Practices for Outcomes Research Task Force Report. Value Health.

[68] Traoré M.K., Zacharewicz G., Duboz R., Zeigler B. (2019). Modeling and simulation framework for value-based healthcare systems. Simulation.

[69] Gillespie J., McClean S., Garg L., Barton M., Scotney B., Fullerton K. (2016). A multi-phase DES modelling framework for patient-centred care. J. Oper. Res. Soc..

[70] Young T., Soorapanth S., Wilkerson J., Millburg L., Roberts T., Morgareidge D. (2018). The costs and value of modelling-based design in healthcare delivery: Five case studies from the US. Health Syst..

[71] Matta M.E., Patterson S.S. (2007). Evaluating multiple performance measures across several dimensions at a multi-facility outpatient center. Health Care Manag. Sci..

[72] Kovalchuk S.V., Funkner A.A., Metsker O.G., Yakovlev A.N. (2018). Simulation of patient flow in multiple healthcare units using process and data mining techniques for model identification. J. Biomed. Inform..

[73] Celano G., Costa A., Fichera S., Tringali G. (2012). Linking Six Sigma to simulation: A new roadmap to improve the quality of patient care. Int. J. Health Care Qual. Assur..

[74] Mykoniatis K., Angelopoulou A. (2020). A modeling framework for the application of multi-paradigm simulation methods. Simulation.

[75] Chang Junior J., Lima F., da Silva Fernandes A.M., de Almeida Guardia F., Da Silva V.D., Maccheri G.A. (2019). Computer Simulation Model for Outpatient Clinics in a Brazilian Large Public Hospital Specialized in Cardiology. Braz. J. Oper. Prod. Manag..

[76] Kisliakovskii I., Balakhontceva M., Kovalchuk S., Zvartau N., Konradi A. (2017). Towards a simulation-based framework for decision support in healthcare quality assessment. Procedia Comput. Sci..

[77] Najafzadeh M., Marra C.A., Galanis E., Patrick D.M. (2009). Cost effectiveness of Herpes Zoster Vaccine in Canada. Pharmacoeconomics.

[78] Pradelli L., Eandi M., Povero M., Mayer K., Muscaritoli M., Heller A.R., Fries-Schaffner E. (2014). Cost-effectiveness of omega-3 fatty acid supplements in parenteral nutrition therapy in hospitals: A discrete event simulation model. Clin. Nutr..

[79] Feng W.H., Lou Z., Kong N., Wan H. (2017). A multiobjective stochastic genetic algorithm for the pareto-optimal prioritization scheme design of real-time healthcare resource allocation. Oper. Res. Health Care.

[80] Katsaliaki K., Mustafee N., Taylor S.J.E., Brailsford S. (2009). Comparing conventional and distributed approaches to simulation in a complex supply-chain health system. J. Oper. Res. Soc..

[81] Uriarte A.G., Zúñiga E.R., Moris M.U., Ng A.H. (2017). How can decision makers be supported in the improvement of an emergency department? A simulation, optimization and data mining approach. Oper. Res. Heath Care.

[82] Xiong W., Bair A., Sandrock C., Wang S., Siddiqui J., Hupert N. (2012). Implementing telemedicine in medical emergency response: Concept of operation for a regional telemedicine hub. J. Med. Syst..

[83] Hussein N.A., Abdelmaguid T.F., Tawfik B.S., Ahmed N.G.S. (2017). Mitigating overcrowding in emergency departments using Six Sigma and simulation: A case study in Egypt. Oper. Res. Health Care.

[84] Vile J.L., Allkins E., Frankish J., Garland S., Mizen P., Williams J.E. (2017). Modelling patient flow in an emergency department to better understand demand management strategies. J. Simul..

[85] De Boeck K., Carmen R., Vandaele N. (2019). Needy boarding patients in emergency departments: An exploratory case study using discrete-event simulation. Oper. Res. Health Care.

[86] Rachuba S., Salmon A., Zhelev Z., Pitt M. (2018). Redesigning the diagnostic pathway for chest pain patients in emergency departments. Health Care Manag. Sci..

[87] Zeinali F., Mahootchi M., Sepehri M.M. (2015). Resource planning in the emergency departments: A simulation-based metamodeling approach. Simul. Model. Pract. Theory.

[88] Lim M.E., Worster A., Goeree R., Tarride J.É. (2013). Simulating an emergency department: The importance of modeling the interactions between physicians and delegates in a discrete event simulation. BMC Med. Inform. Decis. Mak..

[89] Feng Y.Y., Wu I.C., Chen T.L. (2017). Stochastic resource allocation in emergency departments with a multi-objective simulation optimization algorithm. Health Care Manag. Sci..

[90] Choon O.H., Dali Z., Beng P.T., Magdalene C.P.Y. (2014). Uncovering effective process improvement strategies in an emergency department using discrete event simulation. Health Syst..

[91] Gul M., Guneri A.F. (2012). A computer simulation model to reduce patient length of stay and to improve resource utilization rate in an emergency department service system. Int. J. Ind. Eng. Theory Appl. Pract..

[92] Bal A., Ceylan C., Taçoğlu C. (2017). Using value stream mapping and discrete event simulation to improve efficiency of emergency departments. Int. J. Healthc. Manag..

[93] Peng Q., Yang J., Strome T., Weldon E., Chochinov A. (2020). Evaluation of physician in triage impact on overcrowding in emergency department using discrete-event simulation. J. Proj. Manag..

[94] Jat M.N., Rafique R.A. (2020). Mass-Casualty Distribution for Emergency Healthcare: A Simulation Analysis. Int. J. Disaster Risk Sci..

[95] Atalan A., Dönmez C.C. (2020). Optimizing experimental simulation design for the emergency departments. Brazilian J. Oper. Prod. Manag..

[96] Valipoor S., Hatami M., Hakimjavadi H., Akçalı E., Swan W.A., De Portu G. (2021). Data-Driven Design Strategies to Address Crowding and Boarding in an Emergency Department: A Discrete-Event Simulation Study. Health Environ. Res. Des. J..

[97] Strauss C., Bildstein G., Efe J., Flacher T., Hofmann K., Huggler M., Stämpfli A., Schmid M., Schmid E., Gehring C. (2021). Optimizing emergency medical service structures using a rule-based discrete event simulation—A practitioner’s point of view. Int. J. Environ. Res. Public Health.

[98] Aboueljinane L., Sahin E., Jemai Z., Marty J. (2014). A simulation study to improve the performance of an emergency medical service: Application to the French Val-de-Marne department. Simul. Model. Pract. Theory.

[99] Marchesi J.F., Hamacher S., Fleck J.L. (2020). A stochastic programming approach to the physician staffing and scheduling problem. Comput. Ind. Eng..

[100] Maulla R.S., Smarta P.A., Harrisb A., Karasnehc A.A.F. (2009). An evaluation of “fast track” in A&E: A discrete event simulation approach. Serv. Ind. J..

[101] Hu X., Barnes S., Golden B. (2018). Applying queueing theory to the study of emergency department operations: A survey and a discussion of comparable simulation studies. Int. Trans. Oper. Res..

[102] Gearhart R.S. (2017). Demand and capacity modelling for acute services using discrete event simulation. Health Syst..

[103] Baril C., Gascon V., Vadeboncoeur D. (2019). Discrete-event simulation and design of experiments to study ambulatory patient waiting time in an emergency department. J. Oper. Res. Soc..

[104] Atalan A., Donmez C.C. (2019). Employment of emergency advanced nurses of Turkey: A discrete-event simulation application. Processes.

[105] Ordu M., Demir E., Tofallis C., Gunal M.M. (2020). A novel healthcare resource allocation decision support tool: A forecasting-simulation- optimization approach. J. Oper. Res. Soc..

[106] Griffiths J.D., Jones M., Read M.S., Williams J.E. (2010). A simulation model of bed-occupancy in a critical care unit. J. Simul..

[107] Mould G., Bowers J., Dewar C., McGugan E. (2013). Assessing the impact of systems modeling in the redesign of an Emergency Department. Health Syst..

[108] Fu X., Presbitero A., Kovalchuk S.V., Krzhizhanovskaya V.V. (2018). Coupling Game Theory and Discrete-Event Simulation for Model-Based Ambulance Dispatching. Procedia Comput. Sci..

[109] Easter B., Houshiarian N., Pati D., Wiler J.L. (2019). Designing efficient emergency departments: Discrete event simulation of internal-waiting areas and split flow sorting. Am. J. Emerg. Med..

[110] Pawar S., Stanam A. (2019). Developing a DEVS-JAVA Model to Simulate and Pre-test Changes to Emergency Care Delivery in a Safe and Efficient Manner. Lect. Notes Comput. Sci. (Incl. Subser. Lect. Notes Artif. Intell. Lect. Notes Bioinform.).

[111] Baia Medeiros D.T., Hahn-Goldberg S., Aleman D.M., O’Connor E. (2019). Planning Capacity for Mental Health and Addiction Services in the Emergency Department: A Discrete-Event Simulation Approach. J. Healthc. Eng..

[112] Tsai J.C.-H., Weng S.-J., Liu S.-C., Tsai Y.-T., Gotcher D.F., Chen C.-H., Chou C.-A., Kim S.-H. (2020). Adjusting Daily Inpatient Bed Allocation to Smooth Emergency Department Occupancy Variation. Healthcare.

[113] Gabriel G.T., Campos A.T., de Lima Magacho A., Segismondi L.C., Vilela F.F., de Queiroz J.A., Montevechi J.A.B. (2020). Lean thinking by integrating with discrete event simulation and design of experiments: An emergency department expansion. PeerJ Comput. Sci..

[114] Onggo B.S.S., Proudlove N.C., D’Ambrogio S.A., Calabrese A., Bisogno S., Levialdi Ghiron N. (2018). A BPMN extension to support discrete-event simulation for healthcare applications: An explicit representation of queues, attributes and data-driven decision points. J. Oper. Res. Soc..

[115] Masterson B.J., Mihara T.G., Miller G., Randolph S.C., Forkner M.E., Crouter A.L. (2004). Using models and data to support optimization of the military health system: A case study in an intensive care unit. Health Care Manag. Sci..

[116] Hasan I., Bahalkeh E., Yih Y. (2020). Evaluating intensive care unit admission and discharge policies using a discrete event simulation model. Simulation.

[117] Davodabadi A., Daneshian B., Saati S., Razavyan S. (2021). Prioritization of patients in ICU: Composite approach of multiple-criteria decision-making and discrete event simulation. Brazilian J. Oper. Prod. Manag..

[118] Zhu Z., Hen B.H., Teow K.L. (2012). Estimating ICU bed capacity using discrete event simulation. Int. J. Health Care Qual. Assur..

[119] Wilson A.M., Reynolds K.A., Verhougstraete M.P., Canales R.A. (2019). Validation of a Stochastic Discrete Event Model Predicting Virus Concentration on Nurse Hands. Risk Anal..

[120] Sun N.Z., Anand P.A., Snell L. (2017). The importance of considering resource’s tasks when modeling healthcare services with discrete-event simulation: An approach using work sampling method. J. Simul..

[121] Baril C., Gascon V., Miller J., Côté N. (2016). Use of a discrete-event simulation in a Kaizen event: A case study in healthcare. Eur. J. Oper. Res..

[122] D’Andrea E., Choudhry N.K., Raby B., Weinhouse G.L., Najafzadeh M. (2020). A bronchial-airway gene-expression classifier to improve the diagnosis of lung cancer: Clinical outcomes and cost-effectiveness analysis. Int. J. Cancer.

[123] Sewell B., Jones M., Gray H., Wilkes H., Lloyd-Bennett C., Beddow K., Bevan M., Fitzsimmons D. (2020). Rapid cancer diagnosis for patients with vague symptoms: A cost-effectiveness study. Br. J. Gen. Pract..

[124] Persson M., Hvitfeldt-Forsberg H., Unbeck M., Sköldenberg O.G., Stark A., Kelly-Pettersson P., Mazzocato P. (2017). Operational strategies to manage non-elective orthopaedic surgical flows: A simulation modelling study. BMJ Open.

[125] Persson M.J., Persson J.A. (2010). Analysing management policies for operating room planning using simulation. Health Care Manag. Sci..

[126] Holm L.B., Dahl F.A., Barra M. (2013). Towards a multimethodology in health care – synergies between Soft Systems Methodology and Discrete Event Simulation. Health Syst..

[127] Reese K., Avansino J., Brumm M., Martin L., Day T.E. (2020). Determining future capacity for an Ambulatory Surgical Center with discrete event simulation. Int. J. Healthc. Manag..

[128] Burns P., Konda S., Alvarado M. (2020). Discrete-event simulation and scheduling for Mohs micrographic surgery. J. Simul..

[129] Baril C., Gascon V., Cartier S. (2014). Design and analysis of an outpatient orthopaedic clinic performance with discrete event simulation and design of experiments. Comput. Ind. Eng..

[130] Standfield L., Comans T., Raymer M., O’Leary S., Moretto N., Scuffham P. (2016). The Efficiency of Increasing the Capacity of Physiotherapy Screening Clinics or Traditional Medical Services to Address Unmet Demand in Orthopaedic Outpatients: A Practical Application of Discrete Event Simulation with Dynamic Queuing. Appl. Health Econ. Health Policy.

[131] Rohleder T.R., Lewkonia P., Bischak D.P., Duffy P., Hendijani R. (2011). Using simulation modeling to improve patient flow at an outpatient orthopedic clinic. Health Care Manag. Sci..

[132] Anderson G.H., Jenkins P.J., McDonald D.A., Van Der Meer R., Morton A., Nugent M., Rymaszewski L.A. (2017). Cost comparison of orthopaedic fracture pathways using discrete event simulation in a Glasgow hospital. BMJ Open.

[133] Montgomery J.B., Linville B.A., Slonim A.D. (2013). Desktop microsimulation: A tool to improve efficiency in the medical office practice. J. Healthc. Qual..

[134] Pongjetanapong K., Walker C., O’Sullivan M., Lovell-Smith M., Furian N. (2019). Exploring trade-offs between staffing levels and turnaround time in a pathology laboratory using discrete event simulation. Int. J. Health Plan. Manag..

[135] Fairley M., Scheinker D., Brandeau M.L. (2018). Improving the efficiency of the operating room environment with an optimization and machine learning model. Health Care Manag. Sci..

[136] Slocum R.F., Jones H.L., Fletcher M.T., McConnell B.M., Hodgson T.J., Taheri J., Wilson J.R. (2020). Improving chemotherapy infusion operations through the simulation of scheduling heuristics: A case study. Health Syst..

[137] Bowers J., Mould G., Marshall C. (2015). Location of services and the impact on healthcare quality: Insights from a simulation of a musculoskeletal physiotherapy service. J. Oper. Res. Soc..

[138] Sandelands E. (1994). Discrete event simulation and pharmacy process re-engineering. Int. J. Health Care Qual. Assur..

[139] Reynolds M., Vasilakis C., McLeod M., Barber N., Mounsey A., Newton S., Jacklin A., Franklin B.D. (2011). Using discrete event simulation to design a more efficient hospital pharmacy for outpatients. Health Care Manag. Sci..

[140] Borgman N.J., Vliegen I.M.H., Boucherie R.J., Hans E.W. (2018). Appointment scheduling with unscheduled arrivals and reprioritization. Flex. Serv. Manuf. J..

[141] Shakoor M., Al-Nasra M., Abu Jadayil W., Jaber N., Abu Jadayil S. (2017). Evaluation of provided services at MRI department in a public hospital using discrete event simulation technique: A case study. Cogent Eng..

[142] Badilla-Murillo F., Vargas-Vargas B., Víquez-Acuña O., García-Sanz-Calcedo J. (2020). Analysis of the installed productive capacity in a medical angiography room through discrete event simulation. Processes.

[143] Shakoor M., Qureshi M.R., Jadayil W.A., Jaber N., Al-Nasra M. (2020). Application of discrete event simulation for performance evaluation in private healthcare: The case of a radiology department. Int. J. Healthc. Manag..

[144] Singla S. (2020). Demand and Capacity Modelling in Healthcare Using Discrete Event Simulation. Open J. Model. Simul..

[145] Zhu Z. (2011). Impact of different discharge patterns on bed occupancy rate and bed waiting time: A simulation approach. J. Med. Eng. Technol..

[146] Monks T., Worthington D., Allen M., Pitt M., Stein K., James M.A. (2016). A modelling tool for capacity planning in acute and community stroke services. BMC Health Serv. Res..

[147] Hulshof P.J.H., Vanberkel P.T., Boucherie R.J., Hans E.W., van Houdenhoven M., van Ommeren J.K.C.W. (2012). Analytical models to determine room requirements in outpatient clinics. OR Spectr..

[148] Sormaz D.N., Malik M. (2018). Data-driven Simulation Modelling for Progressive Care Units in Hospitals. Procedia Manuf..

[149] Rohleder T.R., Bischak D.P., Baskin L.B. (2007). Modeling patient service centers with simulation and system dynamics. Health Care Manag. Sci..

[150] Mans R., Reijers H., Wismeijer D., Van Genuchten M. (2013). A process-oriented methodology for evaluating the impact of IT: A proposal and an application in healthcare. Inf. Syst..

[151] Amir T., Lee B., Woods R.W., Mullen L.A., Harvey S.C. (2019). A Pilot of Data-Driven Modeling to Assess Potential for Improved Efficiency in an Academic Breast-Imaging Center. J. Digit. Imaging.

[152] Almashrafi A., Vanderbloemen L. (2016). Quantifying the effect of complications on patient flow, costs and surgical throughputs. BMC Med. Inform. Decis. Mak..

[153] Attar A., Duru G., Roblin X., Savarieau B., Brunel P., Lamure M., Peyrin-Biroulet L. (2019). Cost savings using a test-based de-escalation strategy for patients with Crohn’s disease in remission on optimized infliximab: A discrete event model study. Dig. Liver Dis..

[154] Kongpakwattana K., Chaiyakunapruk N. (2020). Application of Discrete-Event Simulation in Health Technology Assessment: A Cost-Effectiveness Analysis of Alzheimer’s Disease Treatment Using Real-World Evidence in Thailand. Value Health.

[155] Willis M., Asseburg C., Slee A., Nilsson A., Neslusan C. (2020). Development and Internal Validation of a Discrete Event Simulation Model of Diabetic Kidney Disease Using CREDENCE Trial Data. Diabetes Ther..

[156] Bae S., Karnon J., Crane G., Bessen T., Desai J., Crowe P., Neuhaus S. (2020). Cost-effectiveness analysis of imaging surveillance in stage II and III extremity soft tissue sarcoma: An Australian perspective. Cost Eff. Resour. Alloc..

[157] Dieleman J.M., Myles P.S., Bulfone L., Younie S., van Zaane B., McGiffin D., Moodie M., Gao L. (2020). Cost-effectiveness of routine transoesophageal echocardiography during cardiac surgery: A discrete-event simulation study. Br. J. Anaesth..

[158] Furzer J., Tessier L., Hodgson D., Cotton C., Nathan P.C., Gupta S., Pechlivanoglou P. (2020). Cost-Utility of Early Breast Cancer Surveillance in Survivors of Thoracic Radiation-Treated Adolescent Hodgkin Lymphoma. J. Natl. Cancer Inst..

[159] Huygens S.A., Ramos I.C., Bouten C.V.C., Kluin J., Chiu S.T., Grunkemeier G.L., Takkenberg J.J.M., Rutten-van Mölken M.P.M.H. (2020). Early cost-utility analysis of tissue-engineered heart valves compared to bioprostheses in the aortic position in elderly patients. Eur. J. Health Econ..

[160] Kongnakorn T., Ward A., Roberts C.S., O’Brien J.A., Proskorovsky I., Caro J.J. (2009). Economic evaluation of atorvastatin for prevention of recurrent stroke based on the SPARCL trial. Value Health.

[161] Väätäinen S., Soini E., Peltola J., Charokopou M., Taiha M., Kälviäinen R. (2020). Economic Value of Adjunctive Brivaracetam Treatment Strategy for Focal Onset Seizures in Finland. Adv. Ther..

[162] Geitona M., Stamuli E., Giannakodimos S., Kimiskidis V.K., Kountouris V., Charokopou M., Christou P. (2019). Lacosamide as a first-line treatment option in focal epilepsy: A cost-utility analysis for the Greek healthcare system. J. Med. Econ..

[163] Eldabi T., Paul R.J., Taylor S.J.E. (2000). Simulating economic factors in adjuvant breast cancer treatment. J. Oper. Res. Soc..

[164] Wang H.I., Roman E., Crouch S., Aas E., Burton C., Patmore R., Smith A. (2018). A Generic Model for Follicular Lymphoma: Predicting Cost, Life Expectancy, and Quality-Adjusted-Life-Year Using UK Population–Based Observational Data. Value Health.

[165] Brailsford S.C., Gutjahr W.J., Rauner M.S., Zeppelzauer W. (2007). Combined discrete-event simulation and ant colony optimisation approach for selecting optimal screening policies for diabetic retinopathy. Comput. Manag. Sci..

[166] Ekman M., Lindgren P., Miltenburger C., Meier G., Locklear J.C., Chatterton M. (2012). Lou Cost effectiveness of quetiapine in patients with acute bipolar depression and in maintenance treatment after an acute depressive episode. Pharmacoeconomics.

[167] Hartz S., Getsios D., Tao S., Blume S., Maclaine G. (2012). Evaluating the cost effectiveness of donepezil in the treatment of Alzheimer’s disease in Germany using discrete event simulation. BMC Neurol..

[168] Van Karnebeek C.D.M., Mohammadi T., Tsao N., Sinclair G., Sirrs S., Stockler S., Marra C. (2015). Health economic evaluation of plasma oxysterol screening in the diagnosis of Niemann-Pick Type C disease among intellectually disabled using discrete event simulation. Mol. Genet. Metab..

[169] Brailsford S.C., Harper P.R., Sykes J. (2012). Incorporating human behaviour in simulation models of screening for breast cancer. Eur. J. Oper. Res..

[170] Qin Y., Freebairn L., Atkinson J.A., Qian W., Safarishahrbijari A., Osgood N.D. (2019). Multi-scale simulation modeling for prevention and public health management of diabetes in pregnancy and sequelae. Lect. Notes Comput. Sci. (Incl. Subser. Lect. Notes Artif. Intell. Lect. Notes Bioinform.).

[171] McKinley K.W., Babineau J., Roskind C.G., Sonnett M., Doan Q. (2020). Discrete event simulation modelling to evaluate the impact of a quality improvement initiative on patient flow in a paediatric emergency department. Emerg. Med. J..

[172] Ren Y., Phan M., Luong P., Wu J., Shell D., Barras C.D., Kok H.K., Burney M., Tahayori B., Seah H.M. (2020). Geographic Service Delivery for Endovascular Clot Retrieval: Using Discrete Event Simulation to Optimize Resources. World Neurosurg..

[173] Di Mascolo M., Gouin A. (2013). A generic simulation model to assess the performance of sterilization services in health establishments. Health Care Manag. Sci..

[174] White D.L., Torabi E., Froehle C.M. (2017). Ice-Breaker vs. Standalone: Comparing Alternative Workflow Modes of Mid-level Care Providers. Prod. Oper. Manag..

[175] Ignone G., Mossa G., Mummolo G., Pilolli R., Ranieri L. (2013). Increasing public healthcare network performance by de hospitalization: A patient pathway perspective. Strateg. Outsourc. Int. J..

[176] Swisher J.R., Jacobson S.H., Jun J.B., Balci O. (2001). Modeling and analyzing a physician clinic environment using discrete-event (visual) simulation. Comput. Oper. Res..

[177] Knight V.A., Williams J.E., Reynolds I. (2012). Modelling patient choice in healthcare systems: Development and application of a discrete event simulation with agent-based decision making. J. Simul..

[178] Pan C., Zhang D., Kon A.W.M., Wai C.S.L., Ang W.B. (2015). Patient flow improvement for an ophthalmic specialist outpatient clinic with aid of discrete event simulation and design of experiment. Health Care Manag. Sci..

[179] Lahr M.M.H., Maas W.J., Van Der Zee D.J., Uyttenboogaart M., Buskens E. (2020). Rationale and design for studying organisation of care for intra-arterial thrombectomy in the Netherlands: Simulation modelling study. BMJ Open.

[180] Chalk D., Trent N., Vennam S., McGrane J., Mantle M. (2019). Reducing delays in the diagnosis and treatment of muscle-invasive bladder cancer using simulation modelling. J. Clin. Urol..

[181] Hashim S., Tahar R.M., Abu bakar E.M. (2003). Simulation study for improving patient treatment services. J. ICT.

[182] Cochran J.K., Bharti A. (2006). Stochastic bed balancing of an obstetrics hospital. Health Care Manag. Sci..

[183] Devapriya P., Strömblad C.T.B., Bailey M.D., Frazier S., Bulger J., Kemberling S.T., Wood K.E. (2015). StratBAM: A Discrete-Event Simulation Model to Support Strategic Hospital Bed Capacity Decisions. J. Med. Syst..

[184] White D.L., Froehle C.M., Klassen K.J. (2011). The effect of integrated scheduling and capacity policies on clinical efficiency. Prod. Oper. Manag..

[185] Burns A.S., Santos A., Cheng C.L., Chan E., Fallah N., Atkins D., Dvorak M.F., Ho C., Ahn H., Paquet J. (2017). Understanding Length of Stay after Spinal Cord Injury: Insights and Limitations from the Access to Care and Timing Project. J. Neurotrauma.

[186] Pendharkar S.R., Bischak D.P., Rogers P., Flemons W., Noseworthy T.W. (2015). Using patient flow simulation to improve access at a multidisciplinary sleep centre. J. Sleep Res..

[187] Zhong X., Lee H.K., Williams M., Kraft S., Sleeth J., Welnick R., Hauschild L., Li J. (2018). Workload balancing: Staffing ratio analysis for primary care redesign. Flex. Serv. Manuf. J..

[188] Perez E., Anandhan V., Novoa C. (2020). A Simulation-Based Planning Methodology for Decreasing Patient Waiting Times in Pure Walk-In Clinics. Int. J. Inf. Syst. Serv. Sect..

[189] Gulhane K., Khan A., Joshi R. (2020). Enhancing operational efficiency of hospitals using discrete event simulation. Int. J. Manag..

[190] Das A. (2020). Impact of the COVID-19 pandemic on the workflow of an ambulatory endoscopy center: An assessment by discrete event simulation. Gastrointest. Endosc..

[191] Doneda M., Yalçındağ S., Marques I., Lanzarone E. (2021). A discrete-event simulation model for analysing and improving operations in a blood donation centre. Vox Sang..

[192] McKinley K.W., Chamberlain J.M., Doan Q., Berkowitz D. (2021). Reducing Pediatric ED Length of Stay by Reducing Diagnostic Testing: A Discrete Event Simulation Model. Pediatr. Qual. Saf..

[193] Yemane A., Abrha H., Gidey K. (2021). Performance Measurement and Improvement of Healthcare Service Using Discrete Event Simulation in Bahir Dar Clinic. J. Optim. Ind. Eng..

[194] Fernandez M.B., Herrera M.M., Trejos C., Romero O.R. (2021). Resources allocation in service planning using discrete event simulation. Ing. Univ..

[195] Mohammed M.A., Mohsin S.K., Mohammed S.J. (2021). The Effectiveness of Using Discrete Event Simulation to Optimize the Quality of Service of Outpatient in Iraq: A Case Study. Iraqi J. Ind. Res..

[196] Abdoli M., Bahadori M., Ravangard R., Babaei M., Aminjarahi M. (2021). Comparing 2 Appointment Scheduling Policies Using Discrete-Event Simulation. Qual. Manag. Health Care.

[197] Haddad M.G., Zouein P.P., Salem J., Otayek R. (2016). Case Study of Lean in Hospital Admissions to Inspire Culture Change. EMJ.

[198] Study S.C., Restrepo-morales J.A., Andrés E., Betancur G., Gabriel J., López V. (2019). Customer Service Multichannel Model in a Health Care Service Provider: A Discrete Competitividad y Gestión. Innovar.

[199] Heim J.A., Huang H., Zabinsky Z.B., Dickerson J., Wellner M., Astion M., Cruz D., Vincent J., Jack R. (2015). Design and implementation of a combined influenza immunization and tuberculosis screening campaign with simulation modelling. J. Eval. Clin. Pract..

[200] Parks J.K., Engblom P., Hamrock E., Satjapot S., Levin S. (2011). Designed to fail: How computer simulation can detect fundamental flaws in clinic flow. J. Healthc. Manag..

[201] Dehlendorff C., Kulahci M., Andersen K.K. (2011). Designing simulation experiments with controllable and uncontrollable factors for applications in healthcare. J. R. Stat. Soc. Ser. C Appl. Stat..

[202] Chemweno P., Thijs V., Pintelon L., Van Horenbeek A. (2014). Discrete event simulation case study: Diagnostic path for stroke patients in a stroke unit. Simul. Model. Pract. Theory.

[203] Elliott T.M., Lee X.J., Foeglein A., Harris P.N., Gordon L.G. (2020). A hybrid simulation model approach to examine bacterial genome sequencing during a hospital outbreak. BMC Infect. Dis..

[204] Karnon J. (2003). Alternative decision modelling techniques for the evaluation of health care technologies: Markov processes versus discrete event simulation. Health Econ..

[205] Demir E., Southern D. (2017). Enabling better management of patients: Discrete event simulation combined with the STAR approach. J. Oper. Res. Soc..

[206] Qiao Y., Ran L., Li J. (2019). Optimization of Teleconsultation Using Discrete-Event Simulation from a Data-Driven Perspective. Telemed. e-Health.

[207] Cudney E.A., Baru R.A., Guardiola I., Materla T., Cahill W., Phillips R., Mutter B., Warner D., Masek C. (2019). A decision support simulation model for bed management in healthcare. Int. J. Health Care Qual. Assur..

[208] Demir E., Southern D., Verner A., Amoaku W. (2018). A simulation tool for better management of retinal services. BMC Health Serv. Res..

[209] Peres I.T., Hamacher S., Oliveira F.L.C., Barbosa S.D.J., Viegas F. (2019). Simulation of Appointment Scheduling Policies: A Study in a Bariatric Clinic. Obes. Surg..

[210] Rezaeiahari M., Khasawneh M.T. (2020). Simulation optimization approach for patient scheduling at destination medical centers. Expert Syst. Appl..

[211] Kozlowski D., Worthington D. (2015). Use of queue modelling in the analysis of elective patient treatment governed by a maximum waiting time policy. Eur. J. Oper. Res..

[212] Qureshi S.M., Purdy N., Neumann W.P. (2020). Development of a Methodology for Healthcare System Simulations to Quantify Nurse Workload and Quality of Care. IISE Trans. Occup. Ergon. Hum. Factors.

[213] Dutta D., Parry F., Obaid M., Ramadurai G. (2020). Mechanical thrombectomy in stroke – planning for service expansion using discrete event simulation. Future Healthc. J..

[214] Melman G.J., Parlikad A.K., Cameron E.A.B. (2021). Balancing scarce hospital resources during the COVID-19 pandemic using discrete-event simulation. Health Care Manag. Sci..

[215] Bhosekar A., Ekşioğlu S., Işık T., Allen R. (2021). A discrete event simulation model for coordinating inventory management and material handling in hospitals. Ann. Oper. Res..

[216] Improta G., Guizzi G., Ricciardi C., Giordano V., Ponsiglione A.M., Converso G., Triassi M. (2020). Agile six sigma in healthcare: Case study at santobono pediatric hospital. Int. J. Environ. Res. Public Health.

[217] Gosavi A., Cudney E.A., Murray S.L., Masek C.M. (2016). Analysis of Clinic Layouts and Patient-Centered Procedural Innovations Using Discrete-Event Simulation. EMJ.

[218] Bakker M., Tsui K.L. (2017). Dynamic resource allocation for efficient patient scheduling: A data-driven approach. J. Syst. Sci. Syst. Eng..

[219] Nikakhtar A., Hsiang S.M. (2014). Incorporating the dynamics of epidemics in simulation models of healthcare systems. Simul. Model. Pract. Theory.

[220] Aslani N., Zhang J. (2014). Integration of simulation and DEA to determine the most efficient patient appointment scheduling model for a specific healthcare setting. J. Ind. Eng. Manag..

[221] Bilodeau B.L., Stanford D.A., Goldszmidt M., Appleton A. (2019). Simulated co-location of patients admitted to an inpatient internal medicine teaching unit: Potential impacts on efficiency and physician-nurse collaboration. INFOR Inf. Syst. Oper. Res..

[222] Kalwer M.A., Mari S.I., Memon M.S., Tanwari A., Siddiqui A.A. (2020). Simulation Based Approach for Improving Outpatient Clinic Operations. Mehran Univ. Res. J. Eng. Technol..

[223] Bae K.-H., Jones M., Evans G., Antimisiaris D. (2019). Simulation modelling of patient flow and capacity planning for regional long-term care needs: A case study. Health Syst..

[224] Lamé G., Jouini O., Stal-Le Cardinal J. (2019). Combining Soft Systems Methodology, ethnographic observation, and discrete-event simulation: A case study in cancer care. J. Oper. Res. Soc..

[225] Demirli K., Al Kaf A., Simsekler M.C.E., Jayaraman R., Khan M.J., Tuzcu E.M. (2021). Using lean techniques and discrete-event simulation for performance improvement in an outpatient clinic. Int. J. Lean Six Sigma.

[226] Lam C., Meinert E., Yang A., Cui Z. (2021). Comparison between centralized and decentralized supply chains of autologous chimeric antigen receptor T-cell therapies: A UK case study based on discrete event simulation. Cytotherapy.

[227] England T.J., Harper P.R., Crosby T., Gartner D., Arruda E.F., Foley K.G., Williamson I.J. (2021). Examining the diagnostic pathway for lung cancer patients in Wales using discrete event simulation. Transl. Lung Cancer Res..

[228] Saidani M., Kim H. (2021). A Discrete Event Simulation-Based Model to Optimally Design and Dimension Mobile COVID-19 Saliva-Based Testing Stations. Simul. Healthc. J. Soc. Simul. Healthc..

[229] Zhuhadar L.P., Thrasher E. (2019). Data analytics and its advantages for addressing the complexity of healthcare: A simulated zika case study example. Appl. Sci..

[230] Gonsalves T., Itoh K. (2009). Service optimization with patient satisfaction in healthcare systems. J. Simul..

[231] Zhu Z., Heng B.H., Teow K.L. (2012). Analysis of factors causing long patient waiting time and clinic overtime in outpatient clinics. J. Med. Syst..

[232] Diamant A., Milner J., Quereshy F. (2018). Dynamic Patient Scheduling for Multi-Appointment Health Care Programs. Prod. Oper. Manag..

[233] Swisher J.R., Jacobson S.H. (2002). Evaluating the design of a family practice healthcare clinic using discrete-event simulation. Health Care Manag. Sci..

[234] Bard J.F., Shu Z., Morrice D.J., Wang D.E., Poursani R., Leykum L. (2016). Improving patient flow at a family health clinic. Health Care Manag. Sci..

[235] Almagooshi S. (2015). Simulation Modelling in Healthcare: Challenges and Trends. Procedia Manuf..

[236] Muravev D., Hu H., Rakhmangulov A., Mishkurov P. (2021). Multi-agent optimization of the intermodal terminal main parameters by using AnyLogic simulation platform: Case study on the Ningbo-Zhoushan Port. Int. J. Inf. Manag..

[237] Postema B.F., Haverkort B.R. (2015). An anylogic simulation model for power and performance analysis of data centres. Lect. Notes Comput. Sci. (Incl. Subser. Lect. Notes Artif. Intell. Lect. Notes Bioinform.).

[238] Van Lent W.A., Vanberkel P., Van Harten W.H. (2012). A review on the relation between simulation and improvement in hospitals. BMC Med. Inform. Decis. Mak..

